# CD38 Deficiency Protects the Heart from Ischemia/Reperfusion Injury through Activating SIRT1/FOXOs-Mediated Antioxidative Stress Pathway

**DOI:** 10.1155/2016/7410257

**Published:** 2016-07-31

**Authors:** Xiao-Hui Guan, Xiao-Hong Liu, Xuan Hong, Ning Zhao, Yun-Fei Xiao, Ling-Fang Wang, Ling Tang, Kai Jiang, Yi-Song Qian, Ke-Yu Deng, Guangju Ji, Mingui Fu, Hong-Bo Xin

**Affiliations:** ^1^Institute of Translational Medicine, Nanchang University, Nanchang 330031, China; ^2^National Laboratory of Biomacromolecules, Institute of Biophysics, Chinese Academy of Sciences, Beijing 100101, China; ^3^Department of Basic Medical Science, Shock/Trauma Research Center, School of Medicine, University of Missouri-Kansas City, Kansas City, MO 64108, USA

## Abstract

Ischemia/reperfusion (I/R) injury induces irreversible oxidative stress damage to the cardiac muscle. We previously observed that CD38 deficiency remarkably protects mouse embryonic fibroblasts (MEFs) from oxidative stress-induced injury. However, whether CD38 deficiency protects from I/R injury in the heart is not explored. Here, we showed that the hearts of CD38 deficient mice or wild type mice supplied with exogenous NAD were significantly protected from ischemia/reperfusion injury, seen as reduction of the myocardial infarct sizes when the mice were subjected to 30 min ischemia followed by 24 hours of reperfusion. Consistently, the protection of CD38 deficiency on hypoxia/reoxygenation (H/R) injury was confirmed with a CD38 knockdown H9c2 stable cell line. Furthermore, we observed that knockdown of CD38 remarkably inhibited ROS generation and intracellular Ca^2+^ overloading induced by H/R in H9c2 cells. The FOXO1 and FOXO3 expressions were significantly elevated by H/R injury in CD38 knockdown cells compared with normal H9c2 cells. The cell immunofluorescence assay showed that FOXO1 nuclear translocation was significantly increased in CD38 knockdown H9c2 cells. In addition, we demonstrated that the increase of FOXO1 nuclear translocation was associated with the increased expressions of antioxidant catalase and SOD2 and the attenuated expression of the ROS generation enzyme NOX4. In conclusion, our results provide new evidence that CD38 deficiency protects the heart from I/R injury through activating SIRT1/FOXOs-mediated antioxidative stress pathway.

## 1. Introduction

Myocardial ischemia/reperfusion (I/R) injury occurs when the blood flow to the myocardium is obstructed and followed by the restoration of blood to the ischemic heart [1]. In response to sudden ischemia, coronary vessels dilate to compensate for the low oxygen supply, allowing for maximal oxygen return/recirculation [2]. However, the continuous deficiency of oxygen during ischemia shifts cardiac metabolism toward anaerobic glycolysis, disrupts ATP generation in the mitochondrial oxidative phosphorylation, reduces overall ATP availability, leads to intracellular Na^+^/Ca^2+^ overload, and thus alters ion homeostasis, cardiac contractility, structural organization, and cell death via necrosis and apoptosis [3]. It is reasonable to consider that the rapid and early restoration of blood flow to the ischemic regions prevents further damage. However, numerous studies have observed the reduced cardiac function and even the acceleration of myocardial injury after reperfusion [1, 4]. Cardiac mitochondria have been recognized as an important source of reactive oxygen species (ROS) in the myocardium, considering that a large number of mitochondria reside in the cardiomyocytes to meet a high energy demand [4]. NADPH oxidases (NOX) also contribute to the major production of O_2_^∙−^ and H_2_O_2_ in cardiovascular cell types [3]. Particularly, highly expressed NOX2 and NOX4 isoforms in the heart play an essential role in regulating the development of cardiomyocytes [5]. In addition, ROS mediates the infiltration of neutrophils, which further contribute to the generation of ROS via NOX activation [6].

CD38 was initially identified as a lymphocyte-specific antigen [7] and was later found to be a major NADase in mammalian tissues [8]. As a membrane protein, CD38 contains a single transmembrane domain, a short N-terminal cytoplasmic tail, and a carboxyl-terminal extracellular domain [9]. The carboxyl-terminal extracellular domain performs its enzymatic functions [10, 11]. CD38 is a multifunctional enzyme that has both ADP-ribosyl cyclase and cADPR hydrolase activities, being capable of cleaving NAD^+^ into cADPR and hydrolyzing cADPR to ADPR [10]. Cyclic ADPR is an important intracellular second messenger that participates in Ca^2+^ mobilization and it is involved in regulating multiple physiological functions and pathogenesis including fertilization [12, 13], T-cell activation [14, 15], chemotaxis [16], insulin secretion [17], and airway constriction and asthma [18, 19].

SIRT1 (silent mating type information regulation 2 homolog 1) is a member of the sirtuin family of class III histone deacetylases (HDACs) which utilize NAD^+^ as a substrate. Nicotinamide adenine dinucleotide (NAD) is a key cellular metabolite that is involved in cellular energetic metabolism and plays important roles in many signaling pathways. In particular, NAD is the substrate of CD38 for synthesis of cADPR and CD38 is a crucial regulator of NAD-dependent deacetylase such as SIRT1 which modulates aging and energy metabolism [20]. SIRT1 targets many substrates, particularly the proteins involved in metabolism and stress response [21]. It has been reported that SIRT1 protects the heart from I/R-induced injury through upregulation of antioxidants and downregulation of proapoptotic molecules [21]. FOXO promotes cardiomyocyte survival upon induction by oxidative stress [22]. SIRT1 enhances transcription of some FOXO target genes [23]. In addition, SIRT1 increases FOXO polyubiquitination and degradation [24]. Taken together, these results suggest that there is an overall model in which SIRT1 increases the ability of FOXO to respond to stress through cell cycle arrest and other adaptations but inhibits FOXO-regulated transcription of apoptotic genes.

It had been reported that there were extremely high NAD^+^ levels in many tissues of CD38 knockout mice [8, 25]. Recently, we observed that mouse embryonic fibroblasts (MEFs) from CD38 KO mice were significantly resistant to oxidative stress such as H_2_O_2_-induced injury and hypoxia/reoxygenation- (H/R-) induced injury compared with wild type MEFs [26]. However, the role of CD38 in I/R-induced heart injury was not evaluated. We hypothesize that CD38 deficiency may protect hearts from I/R injury through FOXO-regulated pathway. In the present study, we showed that protection of CD38 deficiency on I/R injury. Using an RNA interference (RNAi) approach, a CD38 knockdown stable cell line was generated in our laboratory. Consistently, CD38 knockdown also protected cells from H/R-induced injury. Furthermore, the protein expressions of FOXO1 and FOXO3 were elevated in the CD38 knockdown cells, in which the alternations of FOXOs were associated with the increased expressions of the downstream antioxidative enzymes such as catalase and SOD2. Meanwhile, the increased expression of NOX4, an enzyme for ROS generation, and the elevation of intracellular Ca^2+^ induced by H/R stimulation were significantly decreased in CD38 knockdown cells. These findings demonstrated that CD38 may aggravate I/R-induced injury in the heart, indicating that CD38 might be a novel target for prevention and treatment of H/R-induced cardiac injury.

## 2. Materials and Methods

### 2.1. In Vivo Myocardial I/R Model

Male CD38 KO and WT mice at the age of 8–12 weeks in C57BL/6 background were used for this study. Mice were randomly assigned to 5 groups (6 mice for each group): (1) WT sham operation group; (2) WT myocardial I/R group; (3) WT myocardial I/R + NAD^+^ treatment group (NAD^+^ of 2 mg/10 g, IP); (4) CD38 KO sham operation group; (5) CD38 KO myocardial I/R group. The mice were anesthetized with pentobarbital sodium (50 mg/kg), and then a 20 G intravenous catheter was inserted and they were ventilated with a rodent ventilator. A left thoracotomy was performed. LAD coronary artery was visualized and ligated using 6-0 silk suture around fine PE-10 tubing with a slip knot. Mice were subjected to 30 minutes of LAD ischemia followed by 24 h of reperfusion. The infarct areas were determined by Evans blue and TTC staining. At the end of a 24 h reperfusion, the mice were anesthetized, the LAD was reoccluded at the previous ligation, and 1 mL of 2% Evans blue (Sigma-Aldrich, St. Louis, MO) was injected. The heart was quickly excised, immediately frozen, and sliced. Sections were then incubated in a 1% TTC (Sigma-Aldrich) solution for 20 min and digitally photographed by Nikon 1500. The left ventricular area, area at risk (AAR), and infarct area were determined by computerized planimetry using Adobe Photoshop CS4 (Adobe Systems Inc.). All animals were treated in accordance with the Guide for the Care and Use of Laboratory Animals of Nanchang University, and all the experimental protocols were approved by the Ethics Committee of Nanchang University and the experiments were carried out in accordance with the approved guidelines.

### 2.2. Cell Culture and Transfection

H9c2 cells (ATCC, CRL-1446*™*) were cultured in DMEM (Thermo Fisher, Waltham, MA, USA) supplemented with 10% FBS and 100 *μ*g/mL of each of penicillin and streptomycin (Thermo Fisher) at 37°C with 5% CO_2_. The concentration of FBS was reduced to 1% in the medium for 24 h before the induction of H/R injury. A CD38 knockdown H9c2 stable cell line was prepared in our laboratory (unpublished data). Briefly, three different siRNAs sequences targeting rat CD38 mRNA (NM_013127) were designed with the WI siRNA Selection Program and cloned into the retrovirus pSUPER vector (Oligoengine). The resultant three vectors of pSUPER CD38 siRNA1, pSUPER CD38 siRNA2, and pSUPER CD38 siRNA3 and pSUPER scramble siRNA were transfected into Eco-Phoenix packaging cells (provided by the Indiana University Vector Production Facility) by calcium-phosphate precipitation (Promega) according to the manufacturer's instructions. The ecotropic retroviral supernatants were harvested and used for infection of H9c2 cells. The positive clones were picked and expanded after selection with 1 *μ*g/mL puromycin (Sigma-Aldrich) for a week and the levels of CD38 protein in different clones were determined by western blot analysis. The CD38 knockdown siRNA (GGGTAATGCATGACATTAA) and a scramble negative control siRNA (CAGTGCAAGTTGTATCTAA) were used in this study. The transfection reagent used in this study was the SuperFectin II DNA transfection reagent (Pufei Biotech, Shanghai, China). The full length of rat CD38 sequence was cloned into the pcDNA3.0-Flag 1AB vector which was used to transfect the cells that had about 70% confluence. The H/R stimulation was performed after 48 hours of transfection.

### 2.3. H/R and LDH Activity Assay

The cultured medium was changed with hypoxia buffer (NaH_2_PO_4_ 0.9 mmol/L, NaHCO_3_ 6.0 mmol/L, CaCl_2_ 1.0 mmol/L, MgSO_4_ 1.2 mmol/L, HEPES 20 mmol/L, NaCl 98.5 mmol/L, and KCl 10 mmol/L, adjusting to pH = 6.8 with lactic acid) when cells were grown to 80% confluence. The hypoxic condition was generated using a hypoxia chamber. The cells were enclosed in the chamber and flushed with a mixture of gas (95% N_2_ and 5% CO_2_) for 4 hours. The concentration of O_2_ in that chamber was monitored by indicator paper (Oxoid) to ensure it is <1%. The cells were taken out of the chamber after 4 h and the cultured medium was changed with reoxygenation buffer solution (NaH_2_PO_4_ 0.9 mmol/L, NaHCO_3_ 20 mmol/L, CaCl_2_ 1.0 mmol/L, MgSO_4_ 1.2 mmol/L, glucose 5.5 mmol/L, HEPES 20 mmol/L, and NaCl 129.5 mmol/L, adjusting to pH = 7.4). Cells were cultured in normoxic atmosphere for 3 hours. LDH activity was assayed after H/R stimulation according to the instruction from company (Dojindo, Mashiki-machi, Japan).

### 2.4. Western Blot Analysis

Cells were lysed using RIPA buffer (Cell Signaling Technology, Danvers, MA, USA) with proteinase cocktail (Roche Diagnostics, Mannheim, Germany). Proteins were separated by 10–12% SDS-PAGE and transferred to PVDF membrane. The membrane was blocked with 5% nonfat milk for 1 h and incubated at 4°C overnight with the primary antibodies against SOD2 (CST), catalase (CST), and NOX4 (Abcam). The membrane was incubated with HRP-conjugated secondary antibody at 1 : 5000 dilution for 1 h at room temperature and the immune complexes were visualized by chemiluminescence method.

### 2.5. Cellular Immunofluorescence

H9c2 cells with a concentration of 4 × 10^4^ were plated on a 24-well plate with microscope slides (Thermo Fisher) in DMEM supplemented with 10% FBS. Cells were fixed with 4% formaldehyde plus 0.1% Triton X-100 in PBS. Green-conjugated goat anti-rabbit antibody (Thermo Fisher) was used to detect FOXO1 (Santa Cruz Biotechnology, Dallas, Texas, USA, sc11350, rabbit polyclonal IgG, 1 : 100 dilution). Nuclei were stained with DAPI contained in the mounting reagent.

### 2.6. ROS Detection

The intracellular ROS products were measured using H2DCF-DA (Sigma-Aldrich) by monitoring the fluorescent dichlorofluorescein. Cells were incubated with 10 *μ*M H2DCF-DA for 20 min. Then the cells were washed twice with PBS and collected for flow cytometry assay. Five thousand cells were analyzed for each sample. All the steps should be kept away from light.

### 2.7. Apoptosis Detection

The H/R-induced apoptosis assay of H9c2 cells was performed with Annexin V-FIFC apoptosis detection kit (Thermo Fisher). In brief, 3 × 10^5^ H9c2 cells posttreatment were successively stained with Annexin V-FITC and propidium iodide, and then cells were detected by a flow cytometer (Merck Millipore, Darmstadt, Germany). Induced cell apoptosis was presented as percentile of apoptotic cells to total cells.

### 2.8. Real-Time Quantitative PCR

Total RNA was extracted with TRIzol (Thermo Fisher) and subjected to cDNA synthesis using M-MLV Reverse Transcriptase Kit (Thermo Fisher). Expression of the target gene was analyzed by qRT-PCR according to the instruction (Thermo Fisher) and normalized to the reference gene GAPDH. The sequences of the primers are listed below:

 CD38 forward primer 5′-CTGCCAGGATAACTACCGACCT-3′; CD38-reverse primer 5′-CTTTCCCGACAGTGTTGCTTCT-3′; GAPDH-forward primer 5′-AGCCAAAAGGGTCATCATCT-3′; GAPDH-reverse primer 5′-GGGGCCATCCACAGTCTTCT-3′; CAT-forward primer 5′-GAGAAACCCACAGACTCACCT-3′; CAT-reverse primer 5′-GGTCGGTCTTGTAATGGAACT-3′; SOD2-forward primer 5′-GGACAAACCTGAGCCCTAA-3′; SOD2-reverse primer 5′-GCGACCTTGCTCCTTATTG-3′; NOX4-forward primer 5′-AGTCAAACAGATGGGATA-3′; NOX4-reverse primer 5′-TGTCCCATATGAGTTGTT-3′.

### 2.9. Calcium Assay

After H/R stimulation, cells were incubated with 5 *μ*M fluo-3AM (Sigma-Aldrich) at 37°C for 30 min, and then the cells were washed 3 times with HBSS buffer and further incubated for 40 min. Fluorescence was detected using the Millipore Guava flow cytometry assay. The intracellular Ca^2+^ concentrations were determined by measuring the fluorescent density and normalized to negative control.

### 2.10. Cell Viability Assay

Cells were plated in 96-well plates at 1 × 10^4^ cells/well. After 24 h of culture, cells were stimulated with H/R. Cell viability was assessed using cell counting kit-8 (Dojindo, Mashiki-machi, Japan). CCK8 assay is a cell proliferation assay and cytotoxicity assay using WST-8 cleavage. After treatment with CCK-8 at 37°C for 3 h, the absorbance was measured at 450 nm using a microplate reader to quantify the formazan products. The ex527 (Sigma-Aldrich) and LY294002 (Sigma-Aldrich) were used 12 h before H/R stimulation.

### 2.11. Statistical Analysis

Quantitative data are presented as mean ± SE. Differences between experimental groups were analyzed by SPSS, and statistical significance was assessed using Student's independent *t*-test and one-way ANOVA. *p* value < 0.05 was considered statistically significant.

## 3. Results

### 3.1. CD38 Deficient Mice Are Resistant to I/R Injury in Hearts

**Figure 1:**
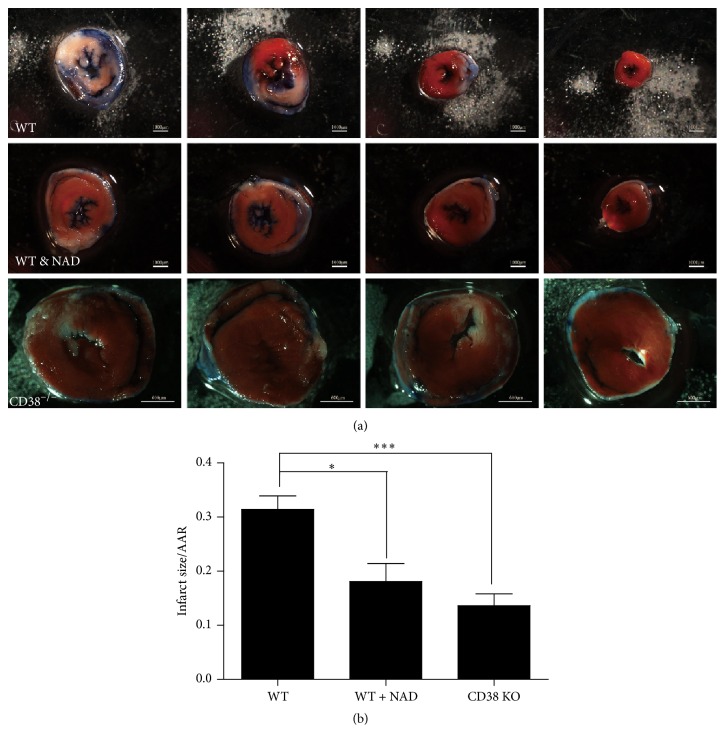
CD38-deficient hearts are resistant to ischemia/reperfusion (I/R) injury in vitro. (a) The images of left ventricular (LV) slices. The mice were subjected to 30 minutes of LAD ischemia followed by 24 h of reperfusion and the infarct area was determined by Evans blue and TTC staining. The nonischemic area is in blue, the area at risk (AAR) is in red, and the infarct area is in white. (First and second row: scale bars = 1000 *μ*m, third row: scale bar = 600 *μ*m.) (b) Quantitative analysis of infarct size/area of CD38 KO and WT hearts after I/R injury. Myocardial injury caused by I/R is significantly attenuated in CD38 KO mice and the WT mice treated with exogenous NAD (2 mg/10 g body weight, IP) compared with WT mice, seeing that the inhibitory percentages of the ratio of infarct size/AAR were reduced from 31.41 ± 5.60% (WT) to 18.07 ± 7.46% (WT + NAD) or 13.63 ± 4.89% (CD38 KO). ^*∗*^ *p* < 0.05 and ^*∗∗*^ *p* < 0.01 and ^*∗∗∗*^ *p* < 0.001, *N* = 6.

To examine the effects of CD38 deficiency on I/R injury in hearts, 12-week-old male CD38 KO and WT mice (20–25 g) were subjected to sham operation or 30 min of LAD coronary artery ligation followed by 24 hours of reperfusion. Myocardial infarction sizes were determined by Evans blue/TTC staining after reperfusion (Figure 1(a)). The mice with sham operation showed almost no infarction after Evans blue/TTC staining which was shown in Figure S1 (see the Supplementary Material available online at http://dx.doi.org/10.1155/2016/7410257). The infarct-to-risk ratios were significantly reduced in CD38 KO mice compared with WT mice (from 31.41 ± 5.60% to 13.63 ± 4.89%, ^*∗∗*^ *p* < 0.01). Consistently, exogenous NAD^+^ supplement (2 mg/10 g body weight, IP) also reduced the infarct-to-risk ratios compared with WT hearts (from 31.41 ± 5.60% to 18.07 ± 7.46%, ^*∗*^ *p* < 0.05, Figure 1(b)). These results indicated that CD38 deficiency might protect hearts from I/R injury.

### 3.2. Knockdown of CD38 Protects Cardiomyocytes from H/R-Induced Cell Death

**Figure 2:**
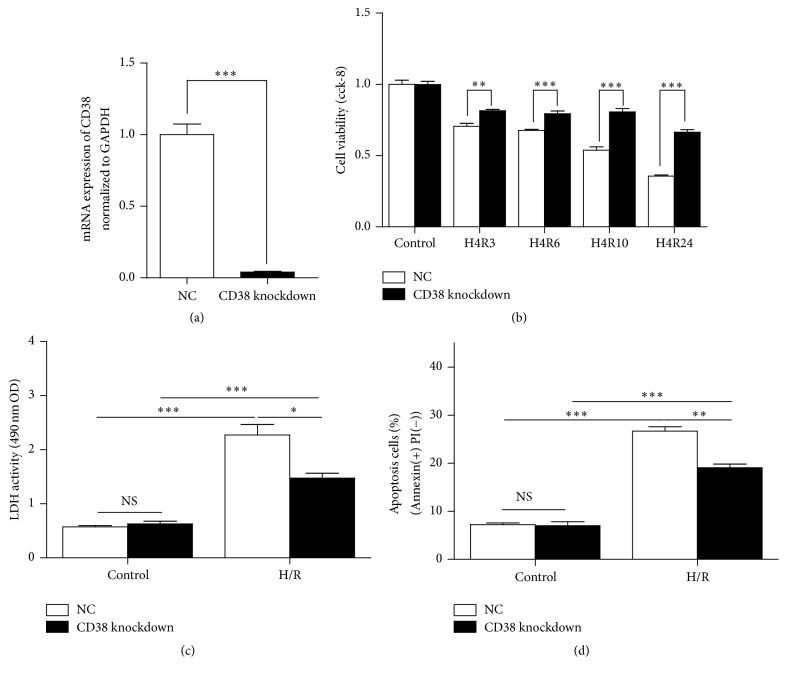
Knockdown of CD38 protects the myocardial cells from hypoxia/reoxygenation- (H/R-) induced death in H9c2 cells. (a) Relative mRNA expressions of CD38 in wild type and CD38 knockdown H9c2 cells. The mRNA expressions of CD38 were determined by real-time PCR and the expression of CD38 was reduced to 5% after gene silence was induced in H9c2 cells. (b) Survival rates of H9c2 cells after 4 h of hypoxia followed different times of reoxygenation. The cell viabilities were examined by CCK8 assay. Although the cell viability of both CD38 knockdown and WT cells was decreased when the cells were treated with H/R, the viability of the myocardial cells in CD38 knockdown cells was much higher than that of WT cells. (c) LDH release in the supernatant of the cell culture after H/R stimulation. The LDH release of CD38 knockdown cells was significantly reduced compared with the normal H9c2 cells. (d) Quantitative analysis of cell apoptosis after H/R stimulation. The apoptosis of myocardial cells was detected with Annexin V-PI staining. The cell apoptosis was attenuated in CD38 gene silenced cells (26.67% ± 0.8969) compared with control cells (19.07% ± 0.7535, *N* = 3, ^*∗*^ *p* < 0.05 and ^*∗∗*^ *p* < 0.01 and ^*∗∗∗*^ *p* < 0.001; NS: no significance).

To confirm the protective effect of CD38 deficiency on cardiomyocytes, a stable H9c2 cell line with CD38 knockdown was used. As shown in Figure 2(a), the mRNA level of CD38 was decreased by 95% in the CD38 knockdown H9c2 cells. To investigate the effects of CD38 knockdown on H/R-induced injury, the cells were subject to 4 hours of hypoxia followed by different times of reoxygenation. The cells with CD38 knockdown had significantly higher survival rate compared with control cells as evaluated by CCK8 (Figure 2(b)). H/R resulted in significant LDH release, which was significantly reduced in the cells with CD38 knockdown (Figure 2(c)). H/R-induced cell apoptosis was also attenuated in the cells with CD38 knockdown as evaluated by Annexin V/PI staining (Figure 2(d)). These results indicated that CD38 deficiency protects the cells from H/R-induced damage in vivo.

To further determine the role of CD38 in H/R-induced injury, CD38 expression vector was introduced into the wild type H9c2 cells or the CD38 knockdown H9c2 stable cell lines. As shown in Figure S2 (see Supplementary Material), the mRNA expressions of CD38 in the cells transfected with CD38 expression vector were much higher than those of the wild type H9c2 cells and the CD38 knockdown cells. The phenotypes of H/R-induced injury were completely reversed by introducing CD38 expression vector in the cells, seen as a significant reduction of the viabilities of the cells overexpressing CD38 with H/R stimulation (Figure S3). In addition, the H/R-induced apoptosis in the cells overexpressing CD38 was markedly increased compared with control cells (Figure S4). All these results further confirmed that the increased expressions of CD38 should aggravate H/R injury in the heart.

### 3.3. Knockdown of CD38 Attenuates H/R-Induced Oxidative Stress

**Figure 3:**
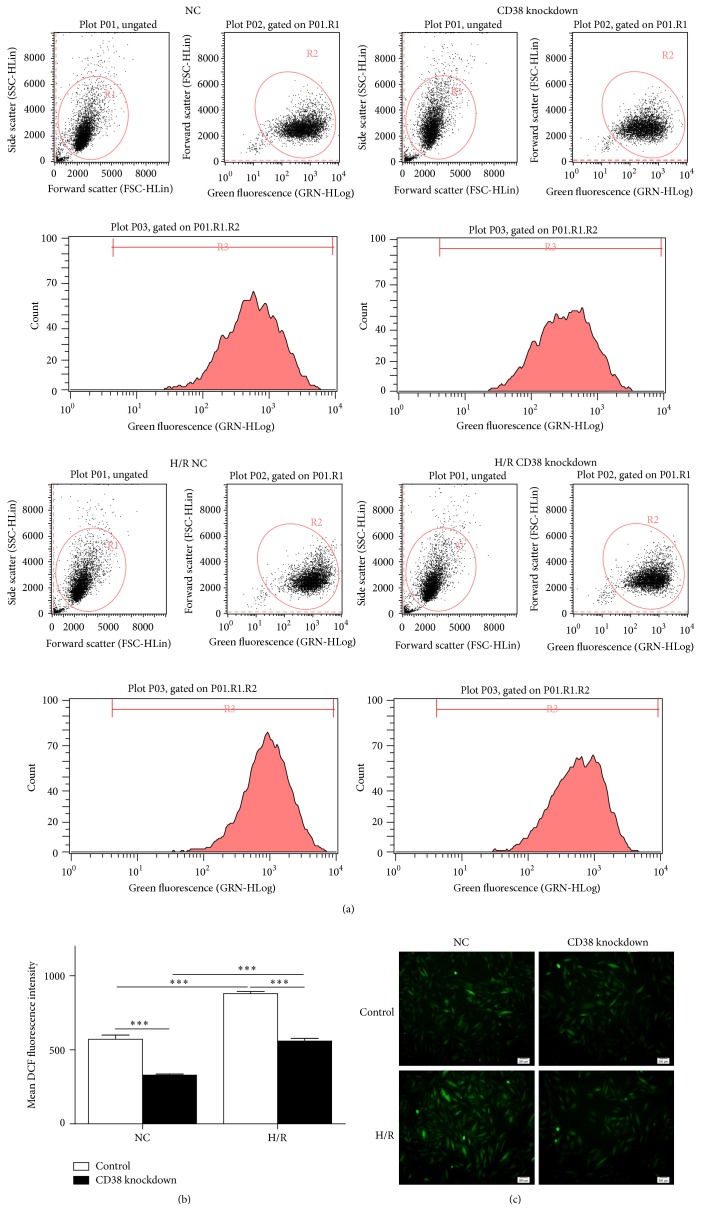
Knockdown of CD38 reduces H/R-induced reactive oxygen species (ROS) generation in H9c2 cells. (a) Representative images of gating plot and peaking plot of intracellular ROS production. The intracellular ROS was stained with CM-H2DCFD and measured by flow cytometry assay. (b) Quantitative analysis of the ROS fluorescence intensity. The mean DCF of fluorescence intensity was summarized from different groups of the cells and the intracellular ROS production was markedly attenuated in CD38 knockdown cells compared with WT cells, regardless of stimulating with or without H/R. (c) The images of ROS detection. Fluorescence images of intracellular ROS in WT or CD38 knockdown H9c2 cells were captured using a fluorescent microscope with CM-H2DCFD staining when the cells were subjected to H/R, and a robust elevation of the intracellular ROS was shown in wild type cells, but not CD38 gene knockdown cells (magnification, ×100 scale bars, 200 *μ*m; ^*∗∗∗*^ *p* < 0.001).

Reactive oxygen species are the major contributors to I/R injury and its excessive production is deleterious to the heart. Next, we examined whether CD38 knockdown reduced H/R-induced ROS generation. The cells were subject to 4 h of hypoxia followed by 3 h of reoxygenation. Then the cells were stained with CM-H2DCFDA for 20 min. As shown in Figure 3, H/R induced a significant increase in ROS generation in control cells, while this effect was greatly attenuated in the cells with CD38 knockdown. These results indicated that CD38 might promote the production of ROS.

### 3.4. The Increased Expressions of FOXOs Protect Hearts against H/R-Induced Oxidative Stress in CD38 Gene Knockdown H9c2 Cells

**Figure 4:**
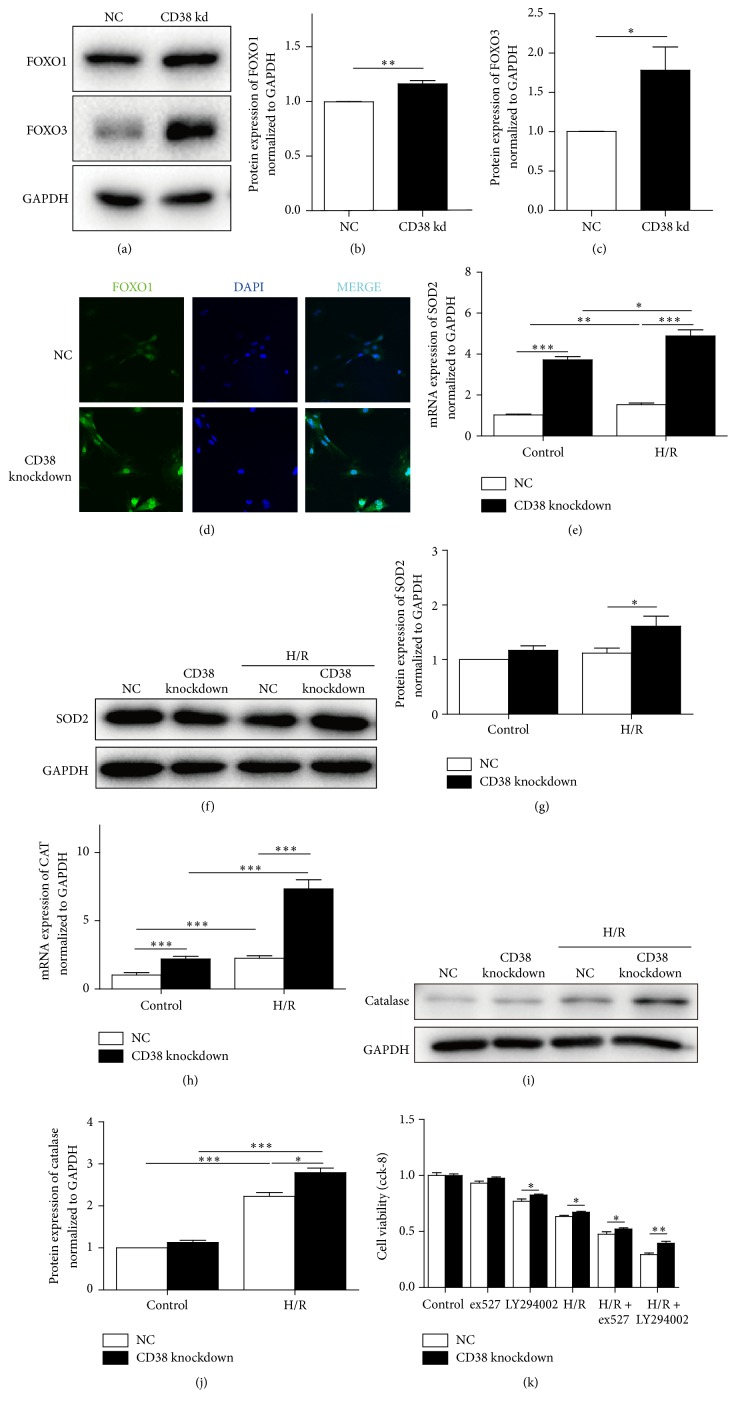
CD38 knockdown protects cells from H/R-induced injury in H9c2 cells through activating FOXOs signaling pathway. (a–c) The protein expressions of FOXO1 and FOXO3 in H9c2 cells. The FOXO proteins were examined by western blot in CD38 knockdown H9c2 cells (a) and the quantitative analysis of the protein expressions of FOXO1 (b) and FOXO3 (c) was performed. (d) The immunofluorescence images of nuclear localization of FOXO1. The H9c2 cells were subjected to immunostaining with anti-FOXO1 antibody (green) and DAPI (blue) (magnification, ×400 scale bars, 20 *μ*m). (e–j) Expressions of SOD2 and catalase (CAT). The mRNA and protein expressions of FOXO1 targeted genes SOD2 and CAT were determined by qPCR (e, h) and western blot with SOD2 (f, g) and CAT (i, j) antibodies, respectively. GAPDH was used as loading control and the data were presented as mean ± SD, *N* = 4, ^*∗*^ *p* < 0.05 and ^*∗∗*^ *p* < 0.01 and ^*∗∗∗*^ *p* < 0.001. (k) Cell viabilities after H/R stimulation with various reagents. The cell viabilities were determined by CCK8 assay after treatment with SIRT1 specific inhibitor EX527 and PI3K inhibiter LY294002 followed with H/R stimulation in H9c2 cells.

Lack of CD38 increases the endogenous activity of the NAD^+^ dependent deacetylation of SIRT1 which promotes FOXO transactivation. It is reasonable for us to consider that CD38 deficiency may protect against H/R-induced oxidative stress through activating SIRT1/FOXO antioxidant pathway. The immune blot results showed that the protein expressions of two antioxidative analogs of forkhead subfamily O, FOXO1 and FOXO3, were elevated in CD38 knockdown H9c2 cells, especially the expression of FOXO3 (Figures 4(a)–4(c)). Although the elevation of FOXO1 is modest, the increased nuclear expression of the protein was increased as shown in Figure 4(d). Consistently, the protein levels of FOXO1 targeting proteins including SOD2 and catalase were markedly increased in the cells with CD38 knockdown. In addition, the protein levels of catalase and SOD2 were also significantly increased in both H/R-stimulated and unstimulated cells with CD38 knockdown (Figures 4(e)–4(j)).

As FOXO transcriptional activity is inhibited by PI3K-Akt signaling [27], whereby PI3K phosphorylation causes Akt to translocate to the nucleus [28], where it phosphorylates FOXO (Thr24, Ser256, and Ser319 of FoxO1; Thr32, Ser253, and Ser315 of FoxO3), the SIRT1 specific inhibitor EX527 (25 *μ*M) and PI3K inhibitor LY294002 (20 *μ*M) were used in the in vitro H/R cell model to clarify the role of CD38 in SIRT1/FOXO antistress signaling pathway. The results in Figure 4(k) showed that both EX527 and LY294002 aggravated the H/R-induced injury in normal H9c2 cells, but these reagents attenuated injury in CD38-si cells, suggesting that inhibition of SIRT1 or FOXO1 activity might have promoted the H/R-induced injury. Taken together, these results suggested that the SIRT1-mediated activation of FOXOs/antioxidative signaling pathway may contribute to CD38 deficiency-mediated protection against H/R-induced oxidative stress and myocardial injury.

### 3.5. CD38 Deficiency Promotes Expression of NOX4 and Decreases Intracellular Ca^2+^ Overloading

**Figure 5:**
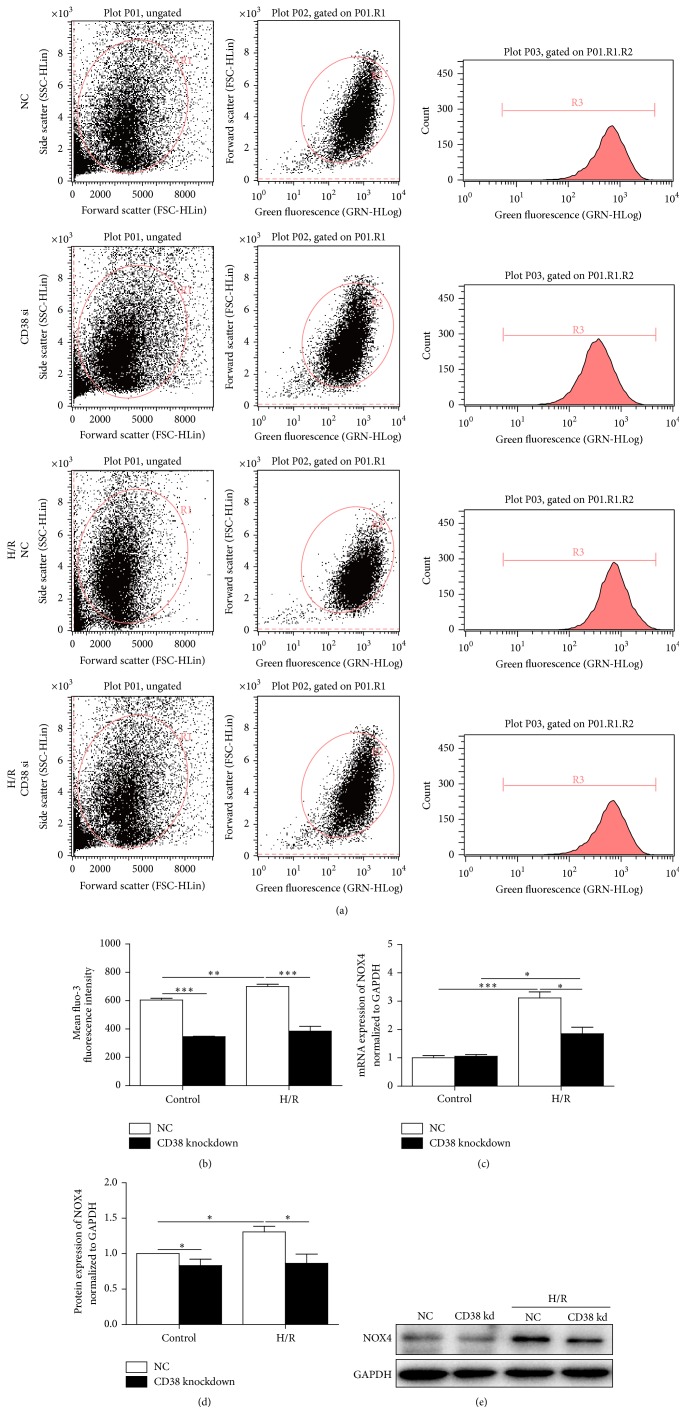
CD38 deficiency promotes expressions of NOX4 and decreases overloading of intracellular Ca^2+^. (a) Representative images of the gating plot and peaking plot of intracellular Ca^2+^ stained with fluo-3AM. The fluorescent dye of fluo-3AM was loaded into myocardial cells and the fluorescent intensity was measured by flow cytometry assay. Although there was a robust elevation of Ca^2+^ in CD38 knockdown and control H9c2 cells after I/R, the concentration of intracellular Ca^2+^ in the CD38 knockdown cells was much lower than that of control cells. (b) Quantification of the mean fluo-3 fluorescence intensity in the different groups of cells. (c–e) Expressions of NOX4 in myocardial cells. The relative mRNA and protein expressions of NOX4 in normal and CD38 knockdown H9c2 cells were determined by qPCR (c) and western blot with NOX4 antibody (d, e), respectively, and GAPDH expression was also detected as a loading control. Data were presented as mean ± SD, *N* = 3, ^*∗*^ *p* < 0.05 and ^*∗∗*^ *p* < 0.01 and ^*∗∗∗*^ *p* < 0.001.

Since the intracellular Ca^2+^ overloading contributes to I/R-induced injury and CD38-generated cADPR is essential for intracellular Ca^2+^ mobilization, we next examined whether the intracellular Ca^2+^ was decreased in the cells with CD38 knockdown in response to H/R stimulation. The concentrations of intracellular Ca^2+^ were detected by fluo-3AM. As shown in Figures 5(a) and 5(b), there was a robust elevation of Ca^2+^ in CD38 knockdown and control H9c2 cells after H/R. However, the concentration of intracellular Ca^2+^ in the cells with CD38 knockdown was much lower than that of control cells. As NOX4 may participate in H/R-induced Ca^2+^ overload, we next examined the effect of CD38 knockdown on the expression of NOX4 after H/R stimulation. As shown in Figures 5(c)–5(e), the H/R-induced mRNA transcription and protein expression of NOX4 in control H9c2 cells, but only modest upregulation, were observed in CD38 knockdown cells.

## 4. Discussion

Myocardial infarction is the major cause of death in the world. Coronary reperfusion therapy has been applying for the management of acute myocardial infarction although myocardial ischemia/reperfusion injury was first observed in the early 1960s [29]. The different clinical manifestations of this injury include myocardial necrosis, arrhythmia, myocardial stunning, and endothelial and microvascular dysfunction including the no-reflow phenomenon. Many studies on experimental models demonstrated that the pathophysiological mechanism of the I/R injury is primarily associated with the production of reactive oxygen species (ROS) and Ca^2+^ overloading in myocardial cells during the processes of ischemia/reperfusion injury [1]. In the present study, we have provided the first evidence that CD38 deficiency protected the heart from I/R injury through inhibiting the production of ROS and attenuating Ca^2+^ overloading in myocardial cells in vivo and in vitro. CD38 is a major NADase in mammalian cells and has numerous functions. As a catalyst, CD38 has both ADP-ribosyl cyclase and cADPR hydrolase activities in which it cleaves NAD to generate cyclic ADP-ribose (cADPR), a putative Ca^2+^ second messenger, and ADPR [19], respectively. In addition, it regulates the activities of enzymes which use NAD^+^ as a substrate such as sirtuins through consuming intracellular NAD [20, 30]. In the present study, we first observed that CD38 deficiency significantly protected the heart from I/R injury in vitro. Meanwhile, the protection was also mimicked when exogenous NAD was injected into the wild type mice, suggesting that the elevation of intracellular NAD mediated by CD38 deficiency may play a key role in the protection of I/R injury in the heart. These results are consistent with our previous observation that CD38 deficiency remarkably protected mouse embryonic fibrocysts (MEFs) from oxidative stress-induced injury [26].

Many studies showed that reactive oxygen species (ROS) generated during early reperfusion lead to extensive oxidative stress to cells, resulting in the loss of cell viability [4], and the reactivation of aerobic metabolism induces a burst of oxidants, particularly O^2−^, which exceeds the endogenous antioxidant capacity [2]. To confirm the protection of CD38 deficiency on I/R injury in myocardial cells, a myocardial cell line with stable knockdown of CD38 gene has been prepared using an RNAi approach. We observed that CD38 deficiency significantly inhibited the generation of ROS in myocardial cells when I/R injury was induced. Meanwhile, we also observed that the cell viability was markedly increased in the myocardial cell line with knockdown of CD38 gene compared with normal cells, indicating that CD38 may promote the cardiac I/R injury in vitro and in vivo.

CD38 is a multifunctional enzyme capable of synthesizing the second messenger, cADPR, and NAADP. However, the major enzymatic activity of CD38 is the hydrolysis of NAD^+^[19]. SIRT1, a NAD-dependent deacetylation enzyme, is activated by CD38 deficiency through elevating intracellular NAD concentrations [20], and it stimulates the transcriptional activity of FOXO [23, 31], which in turn promotes the expressions of its target genes such as SOD and CAT, two potent antioxidants in cardiomyocytes [32]. The forkhead subfamily O is an important mediator of the stress response; it has been demonstrated that FOXOs have unexpected and diverse roles in countering stress, determining cells fate, and regulating energy availability. In addition, an abundance of evidences suggest that three members of the FOXO subfamily—FOXO1, FOXO3, and FOXO4—are critical in maintaining cardiac function and mediating cardiac stresses in the adult (the remaining FOXO family member, FOXO6, is localized in the brain) [32]. To access the mechanism of CD38-mediated promotion of the oxidative stress, the protein expressions of oxidative stress relative signaling molecules FOXO1 and FOXO3 were examined. Interestingly, the expressions of FOXO1 and FOXO3 were significantly increased in CD38 knockdown H9c2 cells compared with the normal cells. We then examined the alternation of the nuclear localization of FOXO1, an oxidative stress relative signaling molecule, in myocardial cells during H/R injury. We observed that lack of CD38 significantly promoted the nuclear translocation of FOXO1 and the expressions of CAT and SOD2 with or without H/R stimulation, consistent with our expectations. Taken together, our results strongly support the hypothesis that CD38 deficiency protects the heart from I/R injury through activating Sirt1/FOXOs-mediated antioxidative stress signaling pathway.

Due to the emerging roles of NAD as a key molecule in multiple signaling pathways and metabolic conditions, any molecule that regulates the synthesis or degradation of this nucleotide may affect multiple physiological processes, such as insulin secretion, control of energy metabolism, neuronal and cardiac cell survival, airway constriction, asthma, aging, and longevity. The discovery of CD38 as the main cellular NADase in mammalian tissues and the characterization of its role on the control of cellular NAD levels indicate that CD38 may serve as a pharmacological target for multiple conditions. On the other hand, poly(ADP-ribose) polymerase (PARP) is another important enzyme for NAD degradation in the NAD metabolism. Depletion of cellular NAD levels from saline between PARP activation and reduced SIRT1 deacetylase activity has been reported in stressed cardiomyocytes, contributing to myocyte cell death during heart failure [33]. However, PARP inhibitors, in addition to their important primary effect of decreasing the activity of nuclear PARP and decreasing NAD(+) and ATP consumption, may reduce ischemia/reperfusion-induced endogenous ROS production and protect the respiratory complexes from ROS induced inactivation [34]. Poly-ADP ribosylation from PARP-1 and other sources of enzymatic poly(ARP-ribose) (PAR) synthesis is associated with cardiac damage following myocardial ischemia [35]. It was also reported that PARP activation and associated cell injury (necrosis) play a crucial role in the intestinal injury, cardiovascular failure, and multiple organ damage associated with resuscitated hemorrhagic shock (HS) [36]. Beside the degradation pathway, the NAD synthesizing pathway is also reported to be beneficial for I/R injury protection. For the overexpression of NAD synthesizing enzyme nicotinamide phosphoribosyltransferase (NAMPT) was reported to protect the heart against I/R injury, significantly decreasing the infarct size and apoptosis [37]. The results from these groups and our lab suggest that any way that increases the content of NAD may play a protective role in myocardial injury.

Our previous study showed that CD38 deficiency protects MEFs from injuries induced by H_2_O_2_ and hypoxia/reoxygenation and demonstrated that the protections are due to reduction of ROS production and intracellular Ca^2+^ overloading in CD38 KO MEFs that were subjected to the oxidative stress-induced injuries [26]. Previous studies have shown that there is a large burst of ROS production after ischemia/reperfusion in many organs [38]. ROS, particularly generated during early reperfusion, would lead to extensive oxidative stress to the cells, resulting in the loss of cell viability [4]. In the present study, we observed that CD38 deficiency not only inhibited ROS generation, but also attenuated intracellular Ca^2+^ overload when I/R or H/R injury was induced in myocardial cells in vivo and in vitro.

Ca^2+^ overload is another key factor for I/R injury in the heart. During ischemia the intracellular H^+^ accumulates as a consequence of anaerobic glycolysis. Once perfusion is restored, H^+^ is transported into the extracellular space in order to normalize the pH in exchange for Na^+^. The resultant increase in intracellular Na^+^ in turn activates the sarcolemmal Na^+^/Ca^2+^ exchanger, resulting in exchange of intracellular Na^+^ with extracellular Ca^2+^. A high rate of 2Na^+^ and 1Ca^2+^ exchange can finally lead to Ca^2+^ overload and cell death [39]. Our results showed that there was a burst elevation of Ca^2+^ after reoxygenation. However, the intracellular Ca^2+^ concentration in CD38 knockdown cells was much lower than that of control cells, indicating that CD38 gene silence attenuated H/R-induced Ca^2+^ overload. NOX4, one of ROS generation enzymes of NOX family, is highly expressed in the heart [40]. We observed that knockdown of CD38 significantly inhibited H/R-induced NOX4 expression in myocardial cells, suggesting that CD38 deficiency protects the heart from I/R-induced injury by both inhibiting oxidative stress and attenuating Ca^2+^ overload induced by ROS in the heart.

Takahashi et al. reported that male CD38 KO mice have a moderate cardiac hypertrophy with an increase of heart weight by 15% compared with WT mice in a range of 3–9 months of age [41]. However, the mice used in our study were 2-3 months old with no observed cardiac hypertrophy [42], and the mice we used were not exactly the same for they were prepared with different knockout strategy [43]. In our previous study, we observed that disruption of CD38 gene enhances cardiac functions by elevating serum testosterone in the male null mice [42]. Furthermore, we found that CD38 gene disruption inhibits the contraction induced by alpha-adrenoceptor stimulation in the mouse aorta and cyclic ADP-ribose-mediated signal transduction system is committed in these responses. Our results suggest that the inhibition of CD38 knockdown-mediated Ca^2+^ signaling pathway may also partially contribute to the protection on the I/R injury in hearts although the CD38-mediated Sirt signaling pathway dominates for the protection.

In conclusion, the results from our present study demonstrated that CD38 deficiency significantly protects the heart from I/R injury in vitro and in vivo, in which the protection is primarily associated with suppressing generation of ROS and overloading of intracellular Ca^2+^ in myocardial cells through activating Sirt1/FOXOs signaling pathway. The findings of this study will broaden our understanding of the roles of CD38 in physiological and pathological conditions in the heart and provide new insights into the mechanisms of the myocardial ischemia/reperfusion injury.

## Supplementary Material

Figure S1. The effects of sham operation on hearts. The Sham-operated animals were subjected to the same surgical procedures, except that the suture was passed under the LAD but not tied. The hearts from wild type and CD38 KO mice with sham operation were stained with evens blue/TTC staining and there was no infarct area in these groups (n = 5). Figure S2. The mRNA expressions of CD38 in H9c2 cells. The mRNA expressions of CD38 were determined by qPCR when a CD38 expression vector was introduced into the normal and CD38 knockdown H9c2 cells. ^***^p<0.001, n = 3. Figure S3. Effects of CD38 overexpression on cell viabilities after H/R stimulation. Cell viability was analyzed by CCK8 assay with various cells after CD38 expression vector was introduced into the cells followed with H/R stimulation. After H/R stimulation, the viabilities of CD38 knockdown H9c2 cells were significantly increased compared with the control cells, whereas the increased CD38 expressions of the normal or CD38 knockdown cells by introducing CD38 expression vector markedly attenuated the cell viabilities. ^*^p<0.05, ^**^p<0.01, ^***^p<0.001, n=5. Figure S4. Effects of CD38 overexpression on apoptosis in H9c2 cells. The H/R induced apoptosis of H9c2 cells were examined by annexinV/PI staining assay after transfected with the CD38 expression vector. The apoptosis in CD38 overexpressing cells was significantly increased compared with normal H9c2 cells after H/R stimulation. ^***^p<0.001, n = 3.
