# Supplementary material for: CD38 Deficiency Protects the Heart from Ischemia/Reperfusion Injury through Activating SIRT1/FOXOs-Mediated Antioxidative Stress Pathway
Source: Oxid Med Cell Longev. 2016 Jul 31;2016:7410257. doi: 10.1155/2016/7410257 (PMC4983367; doi:10.1155/2016/7410257)
Supplement: Supplementary file 1 — Figure S1. The effects of sham operation on hearts. The Sham-operated animals were subjected to the same surgical procedures, except that the suture was passed under the LAD but not tied. The hearts from wild type and CD38 KO mice with sham operation were stained with evens blue/TTC staining and there was no infarct area in these groups (n = 5). Figure S2. The mRNA expressions of CD38 in H9c2 cells. The mRNA expressions of CD38 were determined by qPCR when a CD38 expression vector was introduced into the normal and CD38 knockdown H9c2 cells. ***p<0.001, n = 3. Figure S3. Effects of CD38 overexpression on cell viabilities after H/R stimulation. Cell viability was analyzed by CCK8 assay with various cells after CD38 expression vector was introduced into the cells followed with H/R stimulation. After H/R stimulation, the viabilities of CD38 knockdown H9c2 cells were significantly increased compared with the control cells, whereas the increased CD38 expressions of the normal or CD38 knockdown cells by introducing CD38 expression vector markedly attenuated the cell viabilities. *p<0.05, **p<0.01, ***p<0.001, n=5. Figure S4. Effects of CD38 overexpression on apoptosis in H9c2 cells. The H/R induced apoptosis of H9c2 cells were examined by annexinV/PI staining assay after transfected with the CD38 expression vector. The apoptosis in CD38 overexpressing cells was significantly increased compared with normal H9c2 cells after H/R stimulation. ***p<0.001, n = 3. [file 7410257.f1.pdf]

Supplementary materials:

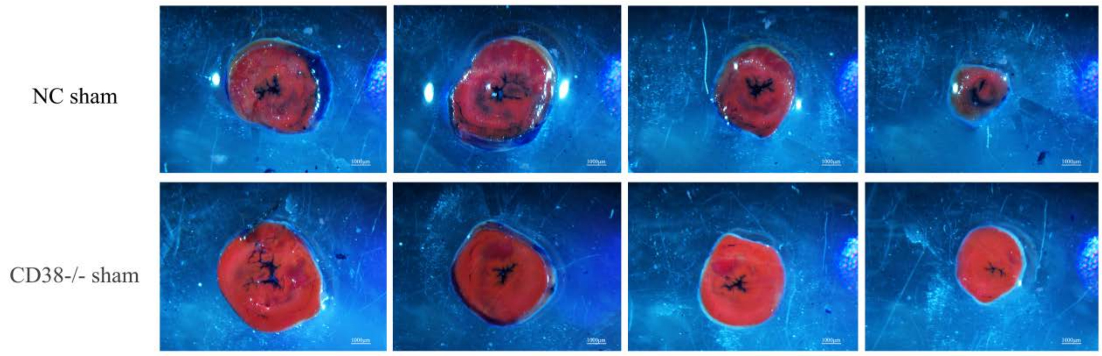

Figure S1. The effects of sham operation on hearts. The hearts from wild type and CD38$^{-/-}$ mice with sham operation were stained with evens blue/TTC staining and there was no infarct area in these groups (n = 5).

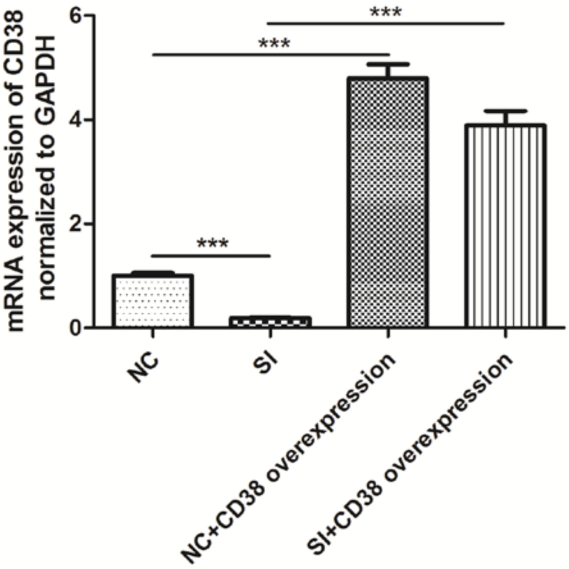

Figure S2. The mRNA expressions of CD38 in H9c2 cells. The mRNA expressions of CD38 were determined by qPCR when a CD38 expression vector was introduced into the normal and CD38 knockdown H9c2 cells. ***p<0.01, n = 3.

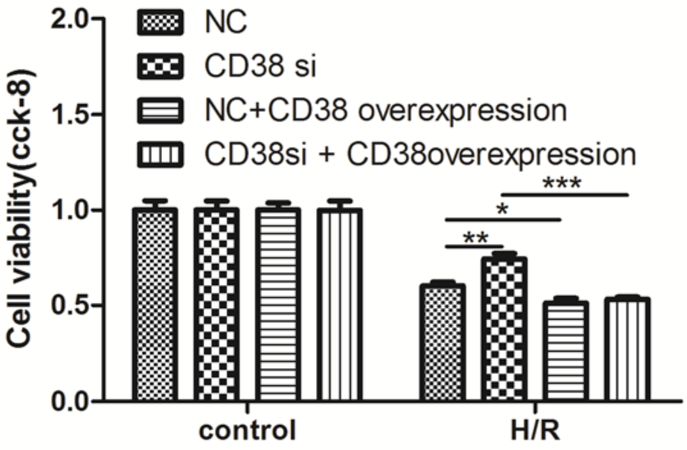

Figure S3. Effects of CD38 overexpressions on cell viabilities after H/R stimulation. Cell viability was analyzed with CCK8 assay with various cells after CD38 expression vector was introduced into the cells followed with H/R stimulation. After H/R stimulation, the viabilities of CD38 knockdown H9c2 cells were significantly increased compared with the control cells, whereas the increased CD38 expressions of the normal or CD38 knockdown cells by introducing CD38 expression vector markedly attenuated the cell viabilities. *p>0.05, **p<0.05, ***p<0.01, N=3.

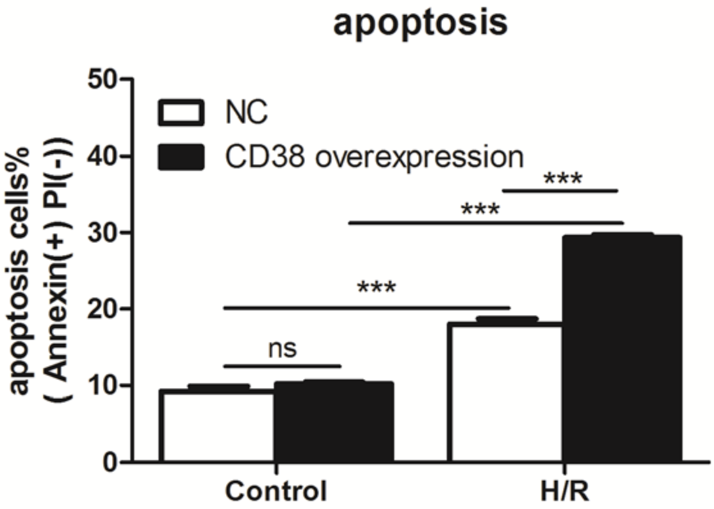

Figure S4. Effects of CD38 overexpressions on apoptosis in H9c2 cells. The H/R induced apoptosis of H9c2 cells were examined by annexinV/PI staining assay after transfected with the CD38 expression vector. The apoptosis in CD38 overexpressing cells was significantly increased compared with normal H9c2 cells after H/R stimulation. ***p<0.01, N = 3.
